# Liver Injury and Metabolic Dysregulation in Largemouth Bass (*Micropterus salmoides*) after Ammonia Exposure

**DOI:** 10.3390/metabo13020274

**Published:** 2023-02-14

**Authors:** Jiahong Zou, Peng Hu, Mengya Wang, Zhenwei Chen, Huan Wang, Xiaolong Guo, Jian Gao, Qingchao Wang

**Affiliations:** 1 College of Fisheries, Huazhong Agricultural University, 1 Shizishan Street, Wuhan 430070, China; 2 Key Laboratory of Fish Conservation and Utilization in the Upper Research of the Yangtze River Sichuan Province, Neijiang Normal University, Neijiang 641000, China

**Keywords:** ammonia exposure, liver, largemouth bass

## Abstract

Elevated environmental ammonia leads to respiratory disorders and metabolic dysfunction in most fish species, and the majority of research has concentrated on fish behavior and gill function. Prior studies have rarely shown the molecular mechanism of the largemouth bass hepatic response to ammonia loading. In this experiment, 120 largemouth bass were exposed to total ammonia nitrogen of 0 mg/L or 13 mg/L for 3 and 7 days, respectively. Histological study indicated that ammonia exposure severely damaged fish liver structure, accompanied by increased serum alanine aminotransferase, aspartate aminotransferase, and alkaline phosphatase activity. RT-qPCR results showed that ammonia exposure down-regulated the expression of genes involved in glycogen metabolism, tricarboxylic acid cycle, lipid metabolism, and urea cycle pathways, whereas it up-regulated the expression of genes involved in gluconeogenesis and glutamine synthesis pathways. Thus, ammonia was mainly converted to glutamine in the largemouth bass liver during ammonia stress, which was rarely further used for urea synthesis. Additionally, transcriptome results showed that ammonia exposure also led to the up-regulation of the oxidative phosphorylation pathway and down-regulation of the mitogen-activated protein kinase signaling pathway in the liver of largemouth bass. It is possible that the energy supply of oxidative phosphorylation in the largemouth bass liver was increased during ammonia exposure, which was mediated by the MAPK signaling pathway.

## 1. Introduction

With the development of intensive aquaculture systems, ammonia has become one of the main environmental pollutants to multiple farmed fish species [1]. Ammonia is rather easy to dissolve in water, and recently high ammonia levels are detected in aquaculture ecosystems due to the accumulation of leftover feed and aquatic animals’ feces [2]. Elevated environmental ammonia levels either impair ammonia excretion or cause a net uptake of ammonia from the environment, resulting in an elevation in body ammonia levels, leading to convulsions and death. Many fish species cannot tolerate high environmental ammonia levels, which may induce irregular osmoregulation capacity, respiration disturbance, oxidative stress, impaired metabolic function, neurotoxicity, immunosuppression, and high mortality in aquaculture [3]. Plenty of studies have been conducted to determine the effects of ammonia toxicity on different fish species. In particular, the gill functions as the main organ for gas exchange, making it more easily attacked by ammonia exposure [4]. Ammonia exposure caused hyperplasia of primary lamellae epithelium and edema of secondary lamellae epithelium both in the model zebrafish [5] and *Osteobrama belangeri* [6]. In golden pompano (*Trachinotus ovatus*), gill oxidative damage caused by acute ammonia stress was proved to be regulated by the hypoxia-inducible factor 1α (HIF-1α)/nuclear factor kappa-B (NF-κb) signaling pathway [7]. Mortality caused by ammonia exposure was mainly attributed to the activation of N-methyl-D-aspartate (NMDA) type glutamate receptor and the influx of excessive Ca^2+^ to induce cell death in the central nervous system [8]. In zebrafish, messenger RNA expressions of glutaminase and glutamate dehydrogenase in the brain were induced, suggesting that ammonia exposure altered glutamate neurotransmitters [9]. Similarly, ammonia stress can disrupt the endocrine and neurotransmitter systems of *Takifugu rubripes* [10].

The liver is a critical hub for numerous physiological processes, including nutrient metabolism [11], immune system support, and breakdown of xenobiotic compounds. In particular, the liver is responsible for the disposal of nitrogenous waste, such as ammonia, from protein degradation in the form of urea metabolism. In fact, the liver is mainly composed of hepatocytes and other liver-resident cells including cholangiocytes, fibroblasts, stellate cells, sinusoidal endothelial cells, and immune cells like Kupffer cells [12]. Hepatocytes are responsible for the metabolism of macronutrients including glucose, lipid, and protein. The ability of the liver to store, synthesize, metabolize, and release glucose is necessary for the postnatal metabolic transition and is maintained throughout the life of an organism. The liver is critical for digestive absorption and performs the uptake, synthesis, packaging, and secretion of lipids and lipoproteins. As a protein synthetic organ, the liver is responsible for 85–90% of circulating protein volume, and the liver also has a high capacity to break down proteins and metabolize the amino acids that comprise them [13]. Additionally, the liver is constantly bombarded by a stream of dietary and commensal bacterial products with inflammatory potential even in healthy individuals, which results in persistent, regulated inflammation [14]. In fish, ammonia exposure has also been reported to affect hepatic structure and nutrient metabolism. For example, ammonia toxicity results in extensive nuclear pycnosis, and a significant increase in eosinophilic lesions were observed in grass carp (*Ctenopharyngodon idellus*) [15]. However, little information is known about the effects of ammonia exposure on hepatic transcriptomic responses.

The largemouth bass (*Micropterus salmoides*) is an important economic freshwater fish, which is native to the United States and is widely distributed around the world [16]. Largemouth bass has become an important economic aquaculture fish species in China whose production reached 702,093 tons in 2021 [17]. The main concern during largemouth bass aquaculture remains liver health, as largemouth bass liver structure was reported to be significantly affected by dietary glucose level [18] and also seriously damaged by *Nocardia seriolae* infection [19]. Till now, only three articles have reported the influences of ammonia on largemouth bass [2,20,21]. Most studies focused on fish behavior and gill function, whereas the ammonia loading on fish liver health and metabolic function remains unclarified. In particular, information on regulatory genes and pathways underlying ammonia stress in largemouth bass is still limited. The knowledge gap impedes the development of breeding or selection of new ammonia-tolerance varieties in largemouth bass. Next-generation, high-throughput method of RNA sequencing (RNA-seq) has been proven to interpret the differentially expressed gene and relevant molecular pathways under certain circumstances. Previous studies have investigated the transcriptomic responses of several fish species including *Ctenopharyngodon idellus* [15], *Coilia nasus* [22], *Cyprinus carpio* [23], and *Paramisgurnus dabryanus* [24] during ammonia stress. Therefore, this study aims to detect the effects of short-term ammonia accumulation stress on liver health and function of largemouth bass, accompanied by the regulatory mechanism via transcriptomic sequencing.

## 2. Materials and Methods

### 2.1. Ethics Statement

All the fish care and experimental procedures were approved by the Animal Experiment Committee of Huazhong Agricultural University (permit number HZAUFI-2017-001).

### 2.2. Fish Rearing and Ammonia Challenge Experiment

Juvenile largemouth bass were bought from a fish farm in Ezhou (Hubei, China) and maintained in the circulating water system in the wet lab of the College of Fisheries at Huazhong Agricultural University. Largemouth bass with an initial weight of 94.78 ± 1.09 g were distributed into 8 experimental tanks (400 L) which were connected to a circulating water system with 15 fish in each tank. Water parameters in the circulating water system were kept constant with the flow at 0.5 L/min and temperature at 26 ± 2 °C. Fish were fed with a diet (crude protein of 47.35%) reported in our previous study [25]. The water pH was maintained at 7.6 ± 0.2, and the dissolved oxygen was maintained at 7.5 ± 0.3 mg/L. Additionally, the photoperiod follows under natural conditions. At the end of the domestication period, four tanks were used for the control group and four tanks were used for the ammonium chloride treatment group. Feeding was stopped one day before ammonia exposure experiments began. Ammonia stock solution was prepared using the chemical ammonium chloride (NH_4_Cl) (Merck reagent grade, Merck and Co., Whitehouse Station, NJ, USA). Referring to previous reports of ammonia exposure in largemouth bass [21] and our preliminary experiment, the concentration of total ammonia nitrogen (TAN) was maintained at 13.0 ± 0.5 mg/L.

After the ammonia challenge for 3 days and 7 days, fish in two groups were anesthetized by 3-Aminobenzoic acid ethyl ester methanesulfonate (MS-222) (100 mg/L; within 2 min) (Sigma, City of Saint Louis, MO, USA). Two fish were taken from each of the four buckets in each group, and blood samples were collected from fish caudal vessels with a heparinized syringe. After perfusing with phosphate-buffered saline (PBS), liver tissues were separated. Partial segments were stored in 4% paraformaldehyde (Servicebio, Wuhan, China) for histopathological assay, and all other liver samples were frozen in liquid nitrogen and then stored at −80 °C before further analysis.

### 2.3. Histological Analysis

After being fixed in 4% paraformaldehyde for at least 24 h, liver tissues were further treated with gradient alcohol for dehydration. Then, after treatment with xylene for transparency, liver tissues were embedded in wax. The obtained tissue paraffin blocks were sectioned at a thickness of 5 μm. The paraffin sections were then stained with hematoxylin-eosin (H. & E.) staining solution (G1001, Servicebio, Wuhan, China). All the stained slides were observed under the optical microscope (Olympus, Tokyo, Japan) and images were collected through CellSens Standard Software.

### 2.4. Assay of Enzyme Activity and Liver Glycogen

The activities of ALT, AST, and ALP in serum were determined and analyzed according to the manufacturer’s instructions (BC1555 & BC1565 & BC2145, Solarbio, Beijing, China). Briefly, ALT catalyzes the transamination of alanine and α-ketoglutaric acid to produce pyruvate and glutamic acid. Then 2,4-dinitrophenylhydrazine solution was added to both terminate the reaction and form phenylhydrazone pyruvate. Phenylhydrazone exhibited a reddish-brown color under alkaline conditions and could be read at 505 nm and calculate enzyme activity. Similarly, AST catalyzes the transamination of α-ketoglutaric acid and aspartic acid to produce glutamic acid and oxaloacetic acid, which further decarboxylated to pyruvate. Pyruvate can react with 2,4-dinitrophenylhydrazine to form 2,4-dinitrophenylhydrazone, which appears brown red under alkaline conditions. The activity of the AST enzyme can be calculated by measuring the change of absorbance at 505 nm. In addition, ALP catalyzes the formation of free phenol from disodium phosphate in an alkaline environment. Phenol reacts with 4-amino-antipyrine and potassium ferricyanide, and has characteristic light absorption at 510 nm. ALP activity was calculated by measuring the absorbance increase rate at 510 nm.

The lactate dehydrogenase (LDH) activities and glycogen content in the liver were also determined with a commercial kit (BC0685 & BC0345, Solarbio, Beijing, China). Briefly, LDH catalyzes NAD+ to oxidize lactic acid to produce pyruvate, and pyruvate further reacts with 2,4-dinitrophenylhydrazine to produce dinitrophenylhydrazone, which is brown-red in an alkaline solution, and the shade of color is proportional to the pyruvate concentration. Similarly, glycogen was extracted with a strong alkaline solution and determined with anthracene chromogenic agent under strong acidic conditions.

### 2.5. RNA Extraction, Library Preparation, and Transcriptome Sequencing

Total RNA was extracted from the liver using TRIzol^®^ Reagent according to the manufacturer’s instructions (Magen, Guangzhou, China). RNA samples were detected based on the A260/A280 absorbance ratio with a Nanodrop ND-2000 system (Thermo Scientific, Waltham, MA, USA), and the RIN of RNA was determined by an Agilent Bioanalyzer 4150 system (Agilent Technologies, Palo Alto, CA, USA). Only qualified samples will be used for library construction. Paired-end libraries were prepared using an ABclonal mRNA-seq Lib Prep Kit (ABclonal, Wuhan, China) following the manufacturer’s instructions. The mRNA was purified from 1 μg total RNA using oligo (dT) magnetic beads followed by fragmentation carried out using divalent cations at elevated temperatures in ABclonal First Strand Synthesis Reaction Buffer. Subsequently, first-strand cDNA was synthesized with random hexamer primers and reverse transcriptase (RNase H) using mRNA fragments as templates, followed by second-strand cDNA synthesis using DNA polymerase I, RNAseH, buffer, and dNTPs. The synthesized double-stranded cDNA fragments were then adapter-ligated for preparation of the paired-end library. Adaptor-ligated cDNA was used for PCR amplification. PCR products were purified (AMPure XP system) and library quality was assessed on an Agilent Bioanalyzer 4150 system. Finally, the library preparations were sequenced on an Illumina Novaseq 6000 and 150 bp paired-end reads were generated.

Data generated from the Illumina platform were used for bioinformatics analysis. All of the analyses were performed using an in-house pipeline from Shanghai Applied Protein Technology. Raw data of fastq format were firstly processed through in-house perl scripts. In this step, remove the adapter sequence and filter out low quality (low quality, the number of lines with a string quality value less than or equal to 25 accounts for more than 60% of the entire reading) and N (N means that the base information cannot be determined) ratio is greater than 5% reads to obtain clean reads that can be used for subsequent analysis. Then, clean reads were separately aligned to the reference genome with orientation mode using HISAT2 software (http://daehwankimlab.github.io/hisat2/) (accessed on 24 October 2022) to obtain mapped reads [26]. FeatureCounts (http://subread.sourceforge.net/) (accessed on 25 October 2022) was used to count the reads numbers gene. Then, fragments per kilobase million (FPKM) of each gene were calculated based on the length of the gene and the reads count mapped to this gene. Differential expression analysis was performed using the DESeq2 (http://bioconductor.org/packages/release/bioc/html/DESeq2.html) (accessed on 25 October 2022); differentially expressed genes (DEGs) with |log2 FoldChange| > 1 and *P*adj < 0.05 were considered to be a significantly different expressed gene [27]. The Gene Ontology (GO) and Kyoto Encyclopedia of Genes and Genomes (KEGG) enrichment analysis of differential genes can explain the functional enrichment of differential genes and clarify the differences between samples at the gene function level. ClusterProfiler R software packages were used for GO function enrichment and KEGG pathway enrichment analysis. When *p* < 0.05, GO or KEGG function was considered as significantly enriched [28].

### 2.6. RT-qPCR Analysis

For cDNA synthesis, 1000 ng of intact RNA was reversed to cDNA with a reverse transcription kit (YEASEN, Shanghai, China) with the concentration detected. The synthesized cDNA was diluted to 200 ng/μL as a template for RT-qPCR. To detect carbohydrate metabolism, the expression of genes related to glycogen synthesis (*ugp2b*, *gys2*), glycogen degradation (*pygl*), gluconeogenesis (*pck1*, *pcxb*), glycolysis (*gck*), TCA cycle pathway (*idh*), and pentose phosphate pathway (*g6pd*) were evaluated. The expression of genes related to lipid synthesis (*fasn*, *acaca*, *aclyb*) and decomposition (*acadl*, *acaa1*, *lpl*) were determined to illustrate the influence on lipid metabolism, and the expression of genes related to the urea cycle (*gs*, *cps3*, *otc*, *ass*, *asl*, and *arg1*) was also detected. The qPCR was performed in Jena qTOWER3G system using the real-time quantitative PCR detection kit EvaGreen 2 × qPCR Master mix (YEASEN, Shanghai, China): pre-denaturation at 95 °C for 5 min; denaturation at 95 °C for 10 s; annealing and extension at 60 °C for 30 s; and PCR reaction step running 40 cycles. After RT-qPCR, melting curves were analyzed to ensure the specificity of the reaction. Using *18s* as the internal reference gene, the relative quantitative data analysis was performed by the 2^−ΔΔCt^ method. RT-qPCR data were analyzed using GraphPad Prism 6. The primers used in the present study are shown in Table 1.

### 2.7. Statistical Analysis

Statistics and analysis of data were performed using GraphPad Prism 6. The data are representative of at least three independent experiments (mean ± SEM). Differences between the two groups were analyzed using Student’s *t*-test, and *p* < 0.05 represented a significant difference (*p* < 0.05 is indicated by *, *p* <0.01 is indicated by **, and *p* < 0.001 is indicated by ***).

## 3. Results

The objective of this study was to explore the effects of ammonia exposure on largemouth bass liver health and function, along with the regulatory mechanism. Histological assay of liver structure and serum ALT, AST, and ALP enzyme activities were conducted to evaluate liver health during ammonia stress. The expression of multiple genes involved in nutrient metabolism was conducted to evaluate liver function during ammonia stress. Moreover, transcriptomic sequencing was conducted to elucidate the regulatory mechanism of ammonia exposure on the largemouth bass liver.

### 3.1. Ammonia Exposure Significantly Affected Liver Health

**Figure 1:**
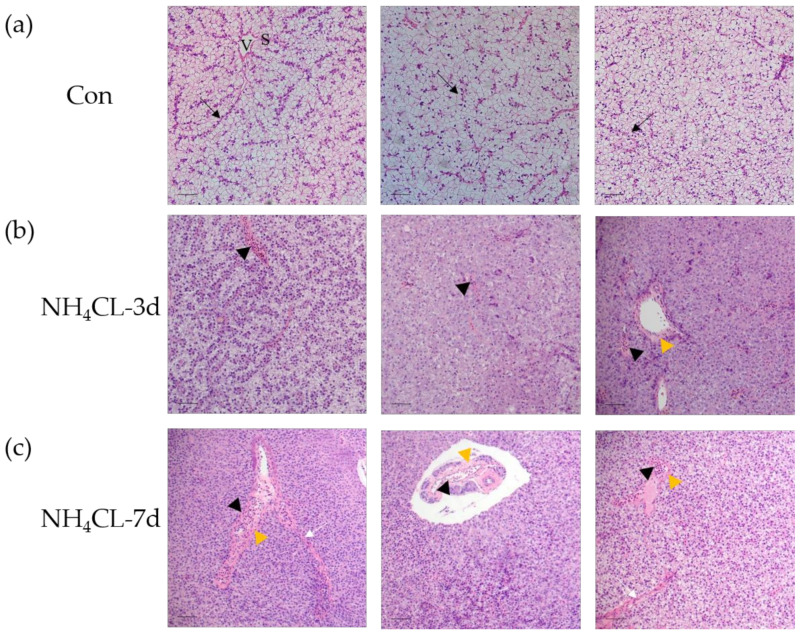
Ammonia exposure affected largemouth bass liver health. Hematoxylin-eosin (H. & e.) staining of paraffin sections of perch liver after ammonia exposure. V: venous tube; S: hepatic sinuses; black arrow: hepatic cord structure; black triangles: infiltration of inflammatory cells; yellow triangle: thickened vascular epithelium; white arrow: hepatic sinus congestion; scale, 50 μm. (**a**) control group; (**b**) ammonia exposure for 3d; (**c**) ammonia exposure for 7 days.

H.&E. staining was conducted to evaluate the influence of ammonia exposure on the largemouth bass liver structure. Results showed that the morphology of hepatocytes, the main cell type of the liver, was complete in the control fish, which were double-layered and radially arranged around the central vein in bundles (Figure 1a). Moreover, the vascular (sinusoids) and biliary (canaliculi) networks exist in the lacunae among hepatocytes. Especially, sinusoids present in the tubular form with narrow sinusoidal capillaries and irregularly shaped sinusoids appearing throughout the interstice between the hepatic plates. Ammonia exposure for 3 and 7 days induced obvious pathological changes in largemouth bass liver structure. As shown in Figure 1a, the liver cord structure was disordered after ammonia exposure for 3 days. The hepatocytes became swollen and the liver was infiltrated with inflammatory cells. Additionally, the hepatic vein endothelium also thickened. With the extension of the ammonia exposure period, hepatocyte injury was aggravated after ammonia exposure for 7 days. Fish hepatocyte swelling was further deepened, along with the increased inflammatory cell infiltration area. Additionally, hepatic cord structure disappeared, while hepatic sinus congestion was observed. Thus, ammonia exposure induced serious pathological changes in the liver of largemouth bass.

**Figure 2:**
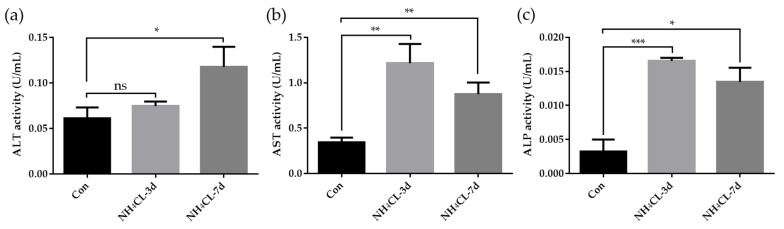
(**a**) Enzyme activities of alanine aminotransferase (ALT), (**b**) aspartate aminotransferase (AST), and (**c**) alkaline phosphatase (ALP) in serum of largemouth bass after ammonia exposure. All data were analyzed with Student’s *t*-test and data were mean ± SEM (*n* = 6). * *p* < 0.05; ** *p* < 0.01; *** *p* < 0.001.

Meanwhile, the enzyme activities of ALT, AST, and ALP activity in the serum of largemouth bass were also determined. As shown in Figure 2, serum ALT activity of largemouth bass was significantly increased after ammonia exposure for 7 days, which was significantly higher than that in the control group (*p* < 0.05). Ammonia exposure for both 3 days and 7 days induced higher serum AST activity and ALP activity. These results along with the histological results confirmed that ammonia exposure seriously affected the liver health of largemouth bass.

### 3.2. Ammonia Exposure Induced Hepatic Metabolic Modulation

**Figure 3:**
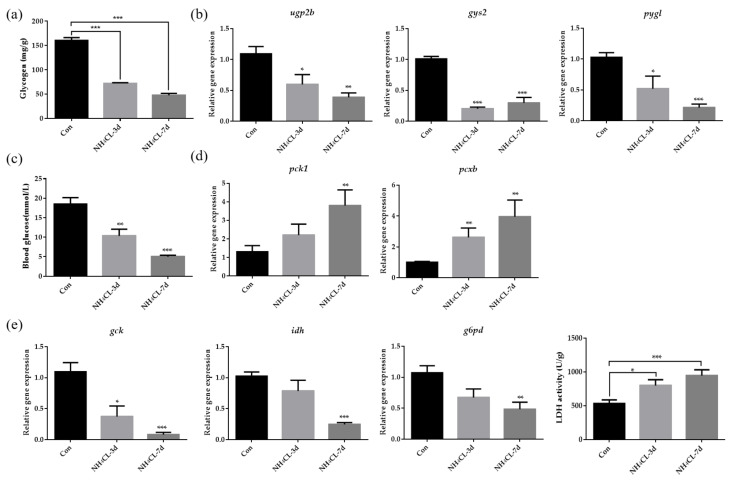
The carbohydrate metabolism in largemouth bass liver after ammonia exposure for 3 days and 7 days was affected. (**a**) Glycogen content in largemouth bass liver. (**b**) The relative expression of genes involved in glycogen metabolism (*ugp2b*, *gys2*, *pygl*) in largemouth bass liver. (**c**) Glucose levels in largemouth bass serum. (**d**) The relative expression of gluconeogenesis-related gene (*pck1*, *pcxb*) in largemouth bass liver. (**e**) The relative expression of genes related to glucose metabolism (*gck*, *idh*, *g6pd*) in largemouth bass liver. Enzyme activity of lactate dehydrogenase activity in largemouth bass liver. All data were analyzed with Student’s *t*-test and data were mean ± SEM (*n* = 6). * *p* < 0.05; ** *p* < 0.01; *** *p* < 0.001.

The glycogen content in largemouth bass liver was evaluated and Figure 3a indicated that ammonia exposure for both 3 and 7 days resulted in decreased glycogen content in largemouth bass liver. Representative genes involved in both glycogen synthesis (*ugp2b* and *gys2*) and glycogen degradation (*pygl*) were also significantly down-regulated after ammonia exposure for both 3 and 7 days (Figure 3b). This was accompanied by decreased serum glucose levels after ammonia exposure (Figure 3c). To be complementary, genes involved in gluconeogenesis including *pck1* and *pcxb* were significantly up-regulated after ammonia exposure (Figure 3d). On the other hand, the expression of *gck*, which is involved in glycolysis from glucose to pyruvate, was significantly decreased after ammonia exposure (Figure 3e). Additionally, *idh*, which is involved in the TCA cycle, was also significantly down-regulated after ammonia exposure. Similarly, the expression of *g6pd*, which is involved in the pentose phosphate pathway, was also significantly down-regulated after ammonia exposure. However, it is interesting that the LDH activity which catalyzes pyruvate to produce lactate was significantly up-regulated after ammonia exposure for both 3 and 7 days (Figure 3e).

**Figure 4:**
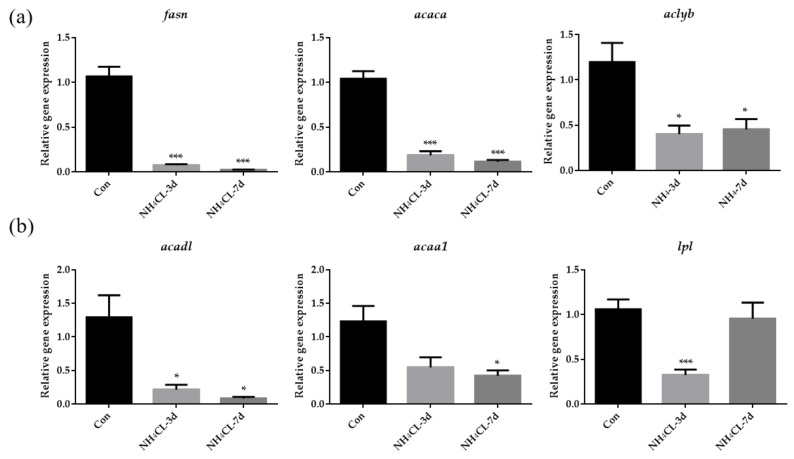
The lipid metabolism in largemouth bass liver after ammonia exposure for 3 days and 7 days was affected. The relative expression of genes involved in lipid synthesis (*fasn*, *acaca*, *aclyb*) (**a**) and lipolysis (*acadl*, *acaa1*, *lpl*) (**b**) in largemouth bass liver after ammonia exposure. All data were analyzed with Student’s *t*-test and data were mean ± SEM (*n* = 6). * *p* < 0.05; *** *p* < 0.001.

Besides carbohydrate metabolism, the hepatic lipid metabolism of largemouth bass after ammonia exposure was also evaluated. As shown in Figure 4a, the expression of *fasn*, *acaca*, and *aclyb*, which are involved in lipogenesis, were significantly down-regulated after ammonia exposure for both 3 days and 7 days. On the other hand, the expression of *acadl,* which catalyzes the first step in fatty acid β-oxidation, was significantly down-regulated after ammonia exposure for both 3 days and 7 days (Figure 4b). Moreover, the expression of *lpl* and *acaa1* was also significantly down-regulated after ammonia exposure for 3 days and 7 days, respectively.

**Figure 5:**
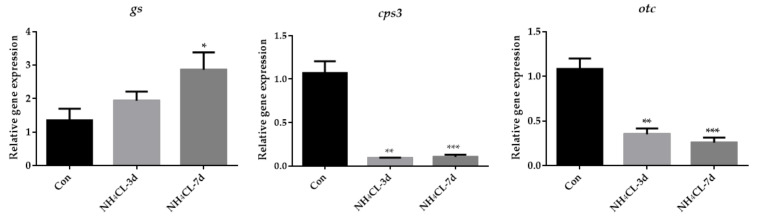

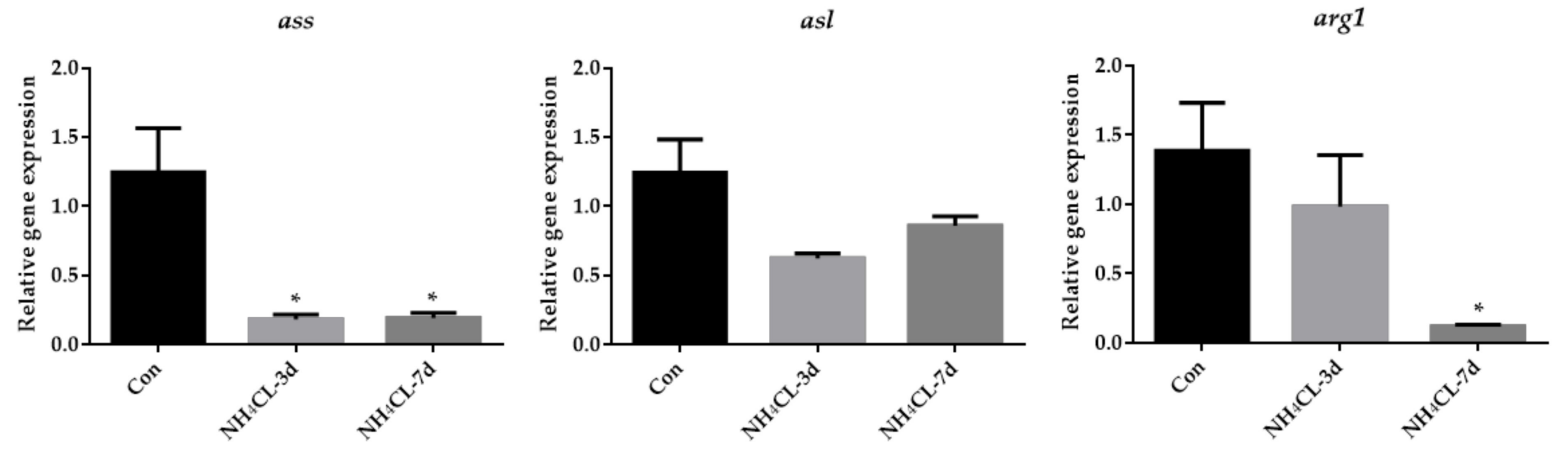
The expression of genes involved in the urea cycle (*gs*, *cps3*, *otc*, *ass*, *asl,* and *arg1*) in largemouth bass liver after ammonia exposure for 3 days and 7 days was affected. All data were analyzed with Student’s *t*-test and data were mean ± SEM (*n* = 6). * *p* < 0.05; ** *p* < 0.01; *** *p* < 0.001.

As the urea cycle in fish liver can transfer ammonia to less-toxic urea, the urea cycle was systemically evaluated. The expression of *gs*, which catalyzes the transfer from ammonia to glutamine, gradually up-regulated with prolonged ammonia exposure and was significantly higher after 7 days of ammonia exposure (Figure 5). However, the expression of *cps III*, *otc*, and *ass* was even down-regulated after ammonia exposure for both 3 days and 7 days. The expression of *asl* showed no significant changes, while the expression of *arg1* was down-regulated after ammonia exposure for 7 days.

### 3.3. Transcriptome Sequencing of Largemouth Bass Liver in Both Control Group and Ammonia Exposure Group

**Figure 6:**
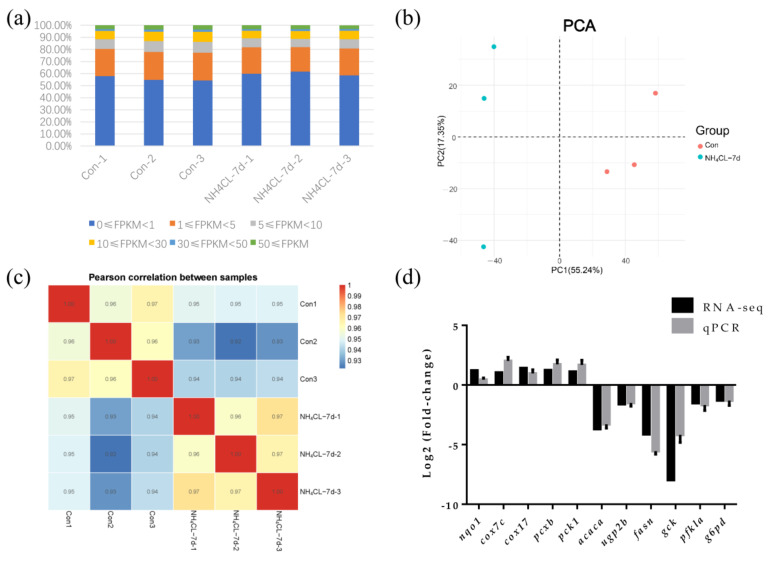
Transcriptomic responses of largemouth bass liver after ammonia exposure for 7d were evaluated by RNA-seq. (**a**) The FPKM value of gene expression levels in two groups. Percentage represents the number of genes with relative abundance in different expression levels and its percentage in the total number of genes. (**b**) The principal component analysis (PCA) diagram shows the clustering of two groups with axes corresponding to two different principal components. Red represents the control group, blue represents the ammonia nitrogen stress samples for 7 days, and 3 replicate samples were conducted for each group. (**c**) Comparative analysis of sample correlation heatmap between control group and ammonia exposure group. (**d**) Eleven differentially expressed genes were randomly selected for qPCR verification.

After removing low-quality reads by fastp from 297,885,618 raw reads, transcriptome sequencing generated 42,896,248 to 54,140,060 clean reads from the six libraries (Table 2). FPKM value can reflect the level of gene expression. So the FPKM value of each gene expression in each sample is shown in Figure 6a. Principal component analysis (PCA) was applied to assess the biological variability and to visualize the distribution of the two groups. The distance between points represents the overall representation difference of the sample. PC1 and PC2 represent different principal components; the numbers in parentheses represent principal component interpretation. The higher the principal component explainability, the greater the overall gene expression difference represented by the distance between sample points in this principal component vector dimension. As shown in the figure, there was no intersection of sample points between the ammonia nitrogen treatment group and the control group, indicating significant differences in gene expression between the two groups (Figure 6b). According to the Pearson correlation between samples, the correlation coefficient of intra-group samples is larger than that of inter-group samples, indicating that intra-group samples have good correlation and repeatability (Figure 6c). In order to confirm the accuracy of RNA-seq results, 12 genes were randomly selected for qRT-PCR assay, whose trends in qRT-PCR assay were consistent with RNA-seq results (Figure 6d). Thus, transcriptome data in the present study are stable and reliable.

### 3.4. Differential Gene Expression Analysis of Largemouth Bass Liver in Both Control Group and Ammonia Exposure Group

**Figure 7:**
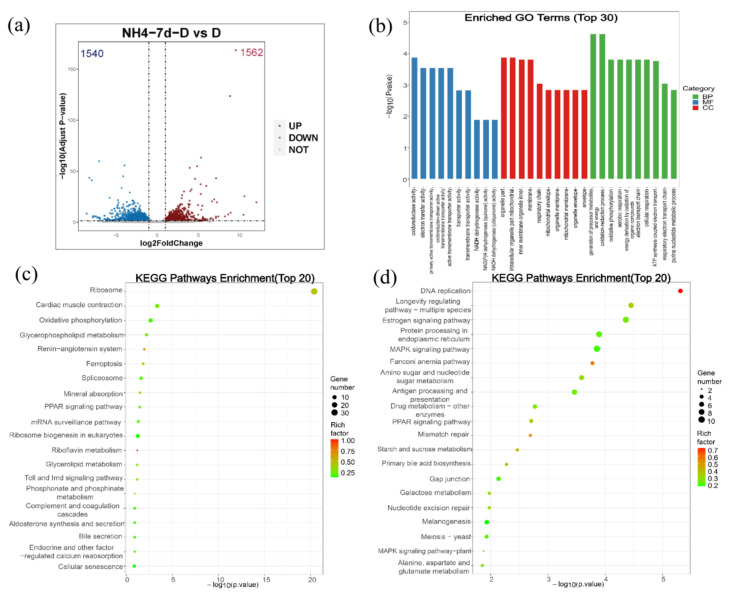
Analysis of the differentially expressed genes (DEGs) and GO enrichment and KEGG pathway analysis of DEGs. (**a**) Volcanic map shows the distribution of DEGs in the liver of largemouth bass after ammonia exposure for 7 days compared with the control group. Gray dots: genes with no significant difference, red dots: up-regulated genes with a significant difference, blue dots: down-regulated genes with a significant difference. (**b**) GO enrichment analysis of DEGs in largemouth bass liver after ammonia exposure for 7 days compared with control group. (**c**) KEGG pathway enrichment analysis of up-regulated DEGs. The size of the bubbles represents number of genes in a pathway, while the color represents rich factor. The X-axis represents −log10 (*p*-value). (**d**) KEGG pathway enrichment analysis of down-regulated DEGs. The size of the bubbles represents number of genes in a pathway, while the color represents rich factor. The X-axis represents −log10 (*p*-value).

For the differential gene expression analysis, *P*adj < 0.05 and |log2FoldChange| > 1 were set as a threshold level to retrieve the differentially expressed genes (DEGs). Compared with the control group, 1562 genes were up-regulated and 1540 genes were down-regulated in the ammonia exposure group (Figure 7a).

For GO enrichment analysis, differentially expressed genes were classified into three major functional categories: molecular functions, cellular components, and biological processes. The top 10 functional items ranked by *p*-value under three GO categories were indicated in Figure 6b. Among them, oxidoreductase activity, electron transfer activity, and primary active transmembrane transporter activity predominated within the molecular functions category. The major terms within the cellular components category were organelle part, intracellular organelle part, and mitochondrial inner membrane. Within the biological process category, the generation of precursor metabolites and energy, oxidation-reduction process, and oxidative phosphorylation were the most well represented subcategories (Figure 7b).

KEGG analyses of both upregulated and downregulated genes were also evaluated, respectively (Figure 7c,d). As shown in Figure 6c, most upregulated genes were mainly enriched in the ribosome, cardiac muscle contraction, oxidative phosphorylation, glycerophospholipid metabolism, and the renin-angiotensin system. Similarly, the top five subcategories of the downregulated genes were mainly enriched in DNA replication, longevity regulating pathway-multiple species, estrogen signaling pathway, the Fanconi anemia pathway, and the MAPK signaling pathway (Figure 7d).

### 3.5. Ammonia Exposure Increased Oxidative Phosphorylation but Inhibited P38 MAPK Pathway

**Figure 8:**
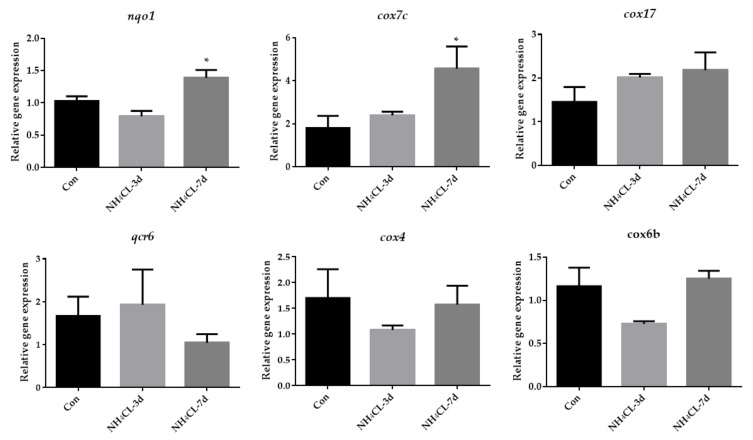
The expression of genes related to oxidative phosphorylation (*nqo1*, *cox7c*, *cox17*, *qcr6*, *cox4*, *cox6b*) in largemouth bass liver after ammonia exposure for 3 days and 7 days was affected. All data were analyzed with Student’s *t*-test and data were mean ± SEM (*n* = 6). * *p* < 0.05.

As mentioned above, oxidative phosphorylation was mainly enriched in KEGG enrichment analysis of up-regulated genes. Thus, oxidative phosphorylation was further examined systemically. As shown in Figure 8, the expression of representative genes involved in the oxidative phosphorylation pathway including *nqo1*, *cox7c*, *cox17*, *qcr6*, *cox4*, and *cox6b* were also evaluated. Results showed that the expression levels of *nqo1* and *cox7c* were significantly up-regulated after ammonia exposure for 7 days, and *cox17* expression level also showed a trend to increase, although it was not significant. The *qcr6*, *cox4,* and *cox6b* did not change significantly.

**Figure 9:**
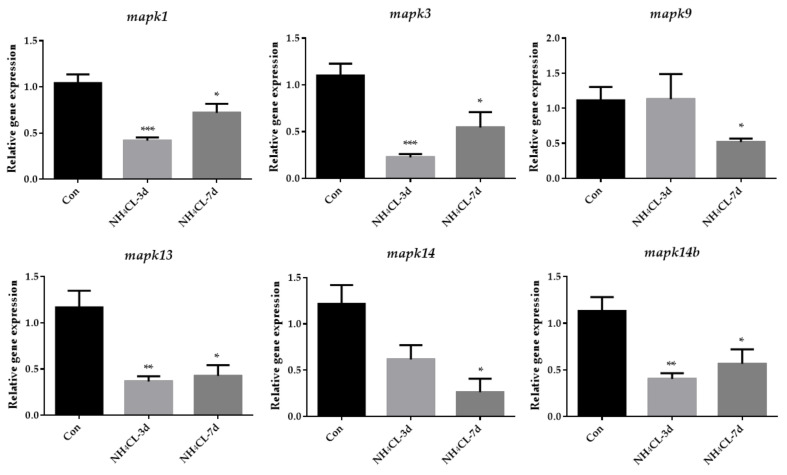
The expression of genes related to MAPK pathway (*mapk1*, *mapk3*, *mapk9*, *mapk13*, *mapk14* and *mapk14b*) in largemouth bass liver after ammonia exposure for 3 days and 7 days was affected. All data were analyzed with Student’s *t*-test and data were mean ± SEM (*n* = 6). * *p* < 0.05; ** *p* < 0.01; *** *p* < 0.001.

Similarly, MAPK signaling pathways were mainly enriched in the KEGG enrichment analysis of down-regulated genes. Then, genes involved in three subfamilies of the MAPK signaling pathway, including extracellular regulated protein kinases (ERK) (*mapk1*, *mapk3*), c-Jun N-terminal kinase (JNK) (*mapk9*), and p38MAPK (*mapk13*, *mapk14* and *mapk14b*) were also evaluated via RT-qPCR. The results showed that the expression of *mapk1* and *mapk3*, which are involved in ERK pathways, were significantly down-regulated after ammonia exposure for 3 days and 7 days (Figure 9). The expression of *mapk9*, which is involved in the JNK pathway, was significantly down-regulated after ammonia exposure for 7 days. Additionally, the expression of *mapk13* and *mapk14b*, which are involved in the p38MAPK pathway, were significantly down-regulated after ammonia exposure for 3 days and 7 days.

## 4. Discussion

In the present study, largemouth bass was treated with ammonia via NH_4_Cl immersion to simulate the ammonia stress in the natural environment. The histological structure of largemouth bass liver was first evaluated via H.&E. staining. In the normal fish liver, hepatocytes were double-layered and radially arranged around the central vein in bundles, with the vascular (sinusoids) and biliary (canaliculi) network distributed in the lacunae among hepatocytes. This is consistent with normal largemouth bass liver morphology reported in our previous study [25] and other studies [30]. Ammonia exposure has been reported to impair the histological features of Nile tilapia liver tissue [31]. Here in this study, ammonia exposure for 3 and 7 days induced obvious pathological changes in largemouth bass liver structure. The liver cord structure was disordered after ammonia exposure for 3 days. The hepatocytes became swollen and the liver was infiltrated with inflammatory cells. Additionally, the hepatic vein endothelium also thickened. With the extension of the ammonia exposure period, hepatocyte injury was aggravated after ammonia exposure for 7 days. Fish hepatocyte swelling was further deepened, along with the increased inflammatory cell infiltration area. Additionally, the hepatic cord structure disappeared, while hepatic sinus congestion was observed. This suggests that ammonia exposure causes structural changes in largemouth bass liver tissue. In order to further evaluate liver health, the enzyme activities of alanine aminotransferase (ALT), aspartate aminotransferase (AST), and alkaline phosphatase (ALP) in serum were also evaluated. ALT and AST are generally present in liver cells (mainly hepatocytes and liver parenchymal cells) and will enter into the blood when the liver is damaged; thus, their activities in serum were labeled as the marker for liver health [32]. In addition, ALP is an important serum analyte, and its elevation is associated with liver disease [33]. Here in this study, serum ALT activity of largemouth bass was significantly increased after ammonia exposure for 7 days, which was significantly higher than that in the control group (*p* < 0.05). Ammonia exposure for both 3 days and 7 days induced higher serum AST and ALP activity. This is similar to earlier studies in other fish species; for example, increased AST and ALT activity was observed in black sea bream (*Acanthopagrus schlegelii*) [34] and grass carp (*Ctenopharyngodon idella*) [35] after exposure to high ammonia concentrations. High ammonia levels have been observed in golden pompano (*Trachinotus ovatus*) [7] resulting in a significant increase in ALP. These results along with the histological results confirmed that ammonia exposure seriously affected the liver health of largemouth bass.

As known, the liver is an indispensable and comprehensive center for the digestion, absorption, metabolism, and purification of various nutrients and substances. In animals, nutrients are absorbed into the blood by the intestine after feeding, and glucose, fructose, and lactose are imported into the liver, among which 60%~70% are converted into glycogen for storage. Liver glycogen can also be decomposed into glucose and converted into energy when the carbohydrate intake is limited [36], which process is controlled by insulin and glucagon [37]. In the present study, ammonia exposure for both 3 and 7 days resulted in decreased glycogen content in the largemouth bass liver, suggesting that glycogen may be decomposed into glucose for energy production during ammonia exposure. Accordingly, the expression of genes related to glycogen synthesis (*ugp2b* and *gys2*) and degradation (*pygl*) was also reduced, indicating high energy expenditure during ammonia exposure and no excess energy to support glycogen synthesis. This was accompanied by decreased serum glucose levels after ammonia exposure. To be compensatory, genes involved in gluconeogenesis including *pck1* and *pcxb* were significantly up-regulated after ammonia exposure. Meanwhile, the expression of *gck*, which is the rate-limiting enzyme in glycolysis from glucose to pyruvate, and the expression of *idh*, which is involved in the TCA cycle, were significantly decreased after ammonia exposure. This is also in accordance with a previous study in pigs in which glycolysis-related genes were also down-regulated during ammonia exposure [38] and in brain cells whose TCA cycle reactions were inhibited during ammonia exposure [39]. Moreover, the pentose phosphate pathway was also inhibited with the significantly down-regulated expression of g6pd after ammonia exposure. Lactate dehydrogenase promotes anaerobic glycolysis by converting pyruvate into lactic acid and exists in the liver, muscle, and other tissue cells. When cells are damaged, the serum LDH level will increase [40]. Therefore, the increase in serum LDH level indicates a certain degree of tissue damage, and LDH activity may be an important biomarker of oxidative stress [41]. In this study, lactate dehydrogenase activity was significantly upregulated after ammonia exposure, suggesting that largemouth bass preferred anaerobic metabolism over aerobic metabolism during ammonia stress. Meanwhile, the elevated serum LDH level also suggested that the liver of largemouth bass might be damaged and caused an oxidative stress response. Besides carbohydrate metabolism, ammonia exposure in yellow catfish has been reported to result in a decrease in overall lipids [42]. Similarly, in our study, the expression of genes involved in both lipogenesis (*fasn*, *acaca,* and *aclyb*) and lipolysis (*acadl*, *acaa1,* and *lpl*) were significantly down-regulated after ammonia exposure. Previous studies have also indicated that during high ammonia exposure, glutamine synthetase (*gs*) can be activated to remove excess ammonia for synthesizing glutamine [43,44]. The activity of *gs* in the liver of marble goby (*Oxyeleotris marmoratus*) increased with the increased ambient ammonia levels, accompanied by glutamine level [45]. In this study, the expression of *gs* in largemouth bass liver increased with the prolonged ammonia exposure period and was significant after ammonia exposure for 7 days compared to the control fish. Thus, largemouth bass may protect itself from ammonia stress by converting NH_3_ into less toxic glutamine. However, the expression of *cps III*, *otc*, *ass*, *asl,* and *arg1* did not increase and even decreased after ammonia exposure. This is similar to studies in mudskipper (*Periophthalmodon schlosseri*) [46], mangrove killifish (*Rivulus marmoratus*) [47], and snakehead (*Channa asiatica*) [48], that they do not use urea cycle as a primary means of detoxification during ammonia exposure. Thus, during ammonia exposure, glutamine is stored in the body after synthesis and can only be used for other anabolic processes when environmental conditions become more favorable [49].

Transcriptome sequencing has been widely used to study the transcriptional response of species under ammonia stress [50,51,52]. However, the transcriptome response of the largemouth bass liver to ammonia-nitrogen stress has rarely been reported. Therefore, in this study, RNA-Seq analysis was performed on largemouth bass liver samples from the control group and those exposed to ammonia for 7 days, in order to reveal the molecular mechanism of largemouth bass liver response to ammonia stress after ammonia nitrogen stress. In this experiment, the transcriptome sequencing generated 42,896,248 to 54,140,060 clean reads and identified 1562 up-regulated genes and 1540 down-regulated genes. The oxidoreduction activity, electric transfer activity, generation of precursor metabolites and energy, oxidation-reduction process, and oxidative physiology of GO enrichment analysis are all related to energy metabolism. This is similar to the changed energy metabolism of Chinese mitten crab during ammonia stress [53]. Besides GO enrichment analysis, KEGG enrichment was also conducted, with the upregulated and downregulated genes enriched, respectively. Especially, the oxidative phosphorylation pathway was enriched in KEGG enrichment analysis of the up-regulated genes during ammonia exposure in this study. As known, oxidative phosphorylation is a metabolic pathway that produces large amounts of ATP [54]. Our further study indicated that the expression of genes involved in oxidative phosphorylation including *nqo1*, *cox7c*, and *cox17* were significantly upregulated after ammonia exposure, indicating that ammonia exposure induced the oxidative phosphorylation to produce energy. This is similar to a study in juvenile *Eriocheir sinensis* which also provide energy through enhanced oxidative phosphorylation under high ammonia stress conditions [55]. On the other hand, the MAPK signaling pathway was enriched in the KEGG enrichment analysis of the down-regulated genes during ammonia exposure in this study. Then, genes involved in three subfamilies of the MAPK signaling pathway, including ERK (*mapk1*, *mapk3*), JNK (*mapk9*), and p38MAPK (*mapk13*, *mapk14* and *mapk14b*) were also evaluated via RT-qPCR. Results showed that all these genes were significantly down-regulated after ammonia exposure. Studies have shown that the activities of p38 MAPK and JNK are necessary for the whole body energy consumption [56,57,58]. P38 MAPK can promote glucose transport in an insulin-independent manner [59] and mice lacking ERK1 have been reported to show resistance to diet-induced obesity [60]. All these indicate that the MAPK signal pathway is related to energy metabolism. Thus, the re-modulated metabolic pathways in the present study may be regulated by the down-regulated MAPK signaling pathways.

In conclusion, ammonia exposure can cause tissue damage to the liver of largemouth bass, increase energy consumption via oxidative phosphorylation, and change the nutritional metabolic pathway of largemouth bass, which process may be regulated by the MAPK signaling pathway. Additionally, ammonia was mainly converted to glutamine in largemouth bass liver during ammonia stress, which was rarely further used for urea synthesis. Future studies could be conducted to explore the reaction of different ammonia dosages on the fish liver to give a more comprehensive view.

## Figures and Tables

**Table 1:** Primers used in the present study.

Gene Name	Abbreviation	Primer Sequence	TM (°C)	Amplicon Size (bp)	Source
18S ribosomal RNA	*18s*	F: GCAAAGCTGAAACTTAAAGGAATTG R: TCCCGTGTTGAGTCAAATTAAGC	58.1 59.5	80	XR_005447146
NADH-quinone oxidoreductase subunit I-like	*nqo1*	F: AAAAATGGCGACGAGGACAC R: TGTCATCACGCATTAAACCAAG	59.1 57.6	110	XM_038733185
cytochrome c oxidase subunit 7C	*cox7c*	F: TGAAGGACCAGGGAAGAACCT R: AAGCCACTGCCAAAGAACAAC	60.4 59.7	87	XM_038704624
cytochrome c oxidase copper chaperone	*cox17*	F: GGCCTCCAAACAAATTAAGCC R: GTCTCTGGACAAGCACAGCA	58.0 60.2	127	XM_038721185
cytochrome b-c1 complex subunit 6	*qcr6*	F: CATGCACGAGACCATTGTGTAGC R: ACAGAAAGGGTGGCATGTTGT	62.3 60.4	197	XM_038718343
cytochrome c oxidase subunit 4 isoform 1	*cox4*	F: GGAAGTCTGTCGTTGGAGGG R: GGGACAGGGCCATACACATAC	60.0 60.2	91	XM_038701371
cytochrome c oxidase subunit 6B1	*cox6b*	F: CTGTCCCATGAGCTGGGTTG R: TGGCGATTTCAGACCTTCCC	60.7 60.0	78	XM_038711198
fatty acid synthase	*fasn*	F: CGGGTTGACCTGGGAAGAAT R: ACTAATCGCTTCCTGCGGAC	59.7 60.2	109	XM_038735140
acetyl-CoA carboxylase alpha	*acaca*	F: TCCCCATCATCACTGGACAC R: AGGCTGCAAATACGGTGGAG	59.1 60.4	114	XM_038709737
ATP citrate lyase b	*aclyb*	F: AGGTCTTCCAGCAGCCAAAG R: GTTCCTCCCGAGAGCATACG	60.2 60.0	125	XM_038735486
acyl-CoA dehydrogenase long chain	*acadl*	F: ACAAACCGCAAAGCGAAGAC R: GGTAGCCGCACATCCTCAAA	60.0 60.4	152	XM_038721438
acetyl-CoA acyltransferase 1	*acaa1*	F: ATCATTTCGGGCCACTTGTCT R: GTCAGGCGTCGTGTCCTTT	60.0 60.3	177	XM_038734444
lipoprotein lipase	*lpl*	F: CATGGCTGGACGGTAACAGG R: GTCAGCCAGTCCACAACGAT	60.7 60.3	107	XM_038715978
lipase, hepatic a	*lipca*	F: CAGTATGGGCTCCTCGGTTTT R: TTGTCACTGCACCTGTAGGC	60.1 60.3	122	XM_038701258
glucokinase (hexokinase 4)	*gck*	F: CCCGAGGGATCAGTGTGTG R: CTTGGTCTCCACCTTCCAGC	59.8 60.3	132	XM_038703172
phosphofructokinase	*pfkla*	F: AGCTGTGACCAGAATGGGC R: CTATCACAGTCCCACCCAGC	60.0 59.8	149	XM_038720351
isocitrate dehydrogenase (NADP(+)) 1	*idh1*	F: AGCACAGGACATCATTCCGC R: CTTGATCTTCTGAGACATCCTGATT	60.8 58.4	87	XM_038721368
dihydrolipoamide S-succinyltransferase	*dlst*	F: TGGCTACACCAAAGGGTCTG R: AGCCAGCTCATTCTTACGGG	59.6 59.8	119	XM_038728954
glucose-6-phosphate 1-dehydrogenase	*g6pd*	F: ACATTCTCTCTCTGCCCGGA R: CGGTTCCAGCCTTTTGTGCT	60.3 61.5	127	XM_038722146
phosphoenolpyruvate carboxykinase 1	*pck1*	F: GGCTGGAAGGTTGAGTGTGT R: CACGTAGGTTGCCTTGGTTG	60.2 59.4	70	XM_038696646
pyruvate carboxylase b	*pcxb*	F: TGGACAAATGCACAGGCAGA R: GAGAGGAAACCGTAGCCTGG	60.2 59.8	150	XM_038731352
phosphorylase, glycogen, liver	*pygl*	F: GATCATCGGGGGAAAGGCTG R: CTTGTTTCCCACCACAGGGT	60.5 60.1	109	XM_038728721
UDP-glucose pyrophosphorylase 2b	*ugp2b*	F: GGTTCAGGAGTACGCATCCC R: ATGATGACGGTGCCCTTGAG	60.1 60.1	120	XM_038737118
glycogen synthase 2	*gys2*	F: GGCCCTACTTCGAACACAACT R: CGGCCAAAATGAACCTGGCA	60.3 61.8	118	XM_038738888
glucan (1,4-alpha-), branching enzyme 1b	*gbe1b*	F: GACTTCAGGAGGAGGTATGAGC R: GCTGCACACCAAAGGTCAAG	59.6 60.0	109	XM_038694127
glutamate-ammonia ligase (glutamine synthase) a	*gs*	F: TTCCGCACCGGAGAATGAG R: TTCCTACTATAACACACCTGGAGA	59.8 58.1	119	XM_038718011
N-acetylglutamate synthase	*nags*	F: CAAGGCTTTTCTCCGTGAAG R: TCACCACCACAGGCTTCATA	57.0 58.6	206	XM_038692344
ornithine transcarbamylase	*otc*	F: GAGTTGCACGGACACAGCTA R: AGGTGAGGAAGTCAGCCAGA	60.3 60.2	170	XM_038720771
argininosuccinate synthetase	*ass*	F: CAGGATAAATGCGGTCAGGT R: GCAAAAAGGTAGGCAATGGA	57.7 56.6	160	XM_038733331
argininosuccinate lyase	*asl*	F: CGTGAAAGCTCTGGAAAAGG R: GCAGGAGCACCAATTAGCTC	57.0 59.0	177	XM_038709368
arginase-1-like	*arg1*	F: GCTGGGTGTGAAGGTGTTTT R: TAGGTGAGTCCTCCCACCAC	58.9 60.3	186	XM_038735677
mitogen-activated protein kinase 1	*mapk1*	F: CTATGGGATGGTGTGCTCTG R: GATCTCTCTCAGGGTGCGTT	57.8 59.2	112	XM_038702794
mitogen-activated protein kinase 3	*mapk3*	F: CAACCACATACTGAGCGTCCT R: TTGGGCTTTTCAGGTAGGGC	60.1 60.3	105	XM_038696984
mitogen-activated protein kinase 9	*mapk9*	F: TCGTGCCTGAAAGACGGAG R: ACCAGCACGGTCTTCTCCC	59.7 61.6	90	XM_038699205
mitogen-activated protein kinase 13	*mapk13*	F: CTGCTTGAGAAGATGCTGGTT R: AGGCTGTCGAAATATGGGTG	58.6 57.7	83	[29]
mitogen-activated protein kinase 14	*mapk14*	F: TTCGATGGAGACGAGATGG R: GAGATGAATGACCGCAGGC	56.4 58.7	161	[29]
mitogen-activated protein kinase 14b	*mapk14b*	F: TCCCTGGCACAGACCACATTG R: CTTGGGCATGTGGGGAAGTG	62.6 61.3	134	XM_038696748

**Table 2:** Quality assessment of the sequencing data.

Sample	Raw_Reads	Clean_Reads	Clean_Bases	Error (%)	Q20 (%)	Q30 (%)	GC (%)
Con-1	46,376,164	45,499,224	6.78 G	0.03	97.82	93.8	47.88
Con-2	51,233,194	50,215,662	7.49 G	0.03	97.59	93.26	48.08
Con-3	43,772,118	42,896,248	6.38 G	0.03	97.8	93.77	48.01
NH_4_Cl-7d-1	55,241,382	54,140,060	8.03 G	0.03	97.65	93.39	46.92
NH_4_Cl-7d-2	50,965,374	50,045,184	7.47 G	0.03	97.59	93.22	47.87
NH_4_Cl-7d-3	50,297,386	49,430,098	7.39 G	0.03	97.76	93.58	46.8

## Data Availability

The data presented in this study are openly available in the NCBI Sequence Read Archive (SRA) under accession number SUB12866725.
